# Rigorous Assessment of Guidelines on COVID-19-Related Thrombotic or Thromboembolic Disease: Implications for Clinical Practice of Prevention, Diagnosis, and Treatment

**DOI:** 10.1155/2021/5513744

**Published:** 2021-09-24

**Authors:** Jingyi Liang, Zhufeng Wang, Jiaxing Xie, Hanwen Liang, Jiamin Liang, Mei Jiang, Shiyue Li

**Affiliations:** ^1^National Clinical Research Center for Respiratory Disease, State Key Laboratory of Respiratory Disease, Guangzhou Institute of Respiratory Health, The First Affiliated Hospital of Guangzhou Medical University, Guangzhou, Guangdong, China; ^2^Department of Allergy and Clinical Immunology, National Clinical Research Center for Respiratory Disease, State Key Laboratory of Respiratory Disease, Guangzhou Institute of Respiratory Health, The First Affiliated Hospital of Guangzhou Medical University, Guangzhou, Guangdong, China; ^3^Guangzhou Medical University, Guangzhou, Guangdong, China

## Abstract

**Purpose:**

Severe COVID-19 patients were prone to develop venous thromboembolism. Unfortunately, to date, there is no evidence of any effective medications for thromboembolism in COVID-19. The management of the disease relies on symptomatic and supportive treatments, giving rise to a variety of guidelines. However, the quality of methodology and clinical recommendations remains unknown.

**Materials and Methods:**

We searched Medline, Cochrane Library, Web of Science, websites of international organizations and medical societies, and gray literature databases. Four well-trained appraisers independently evaluated the quality of eligible guidelines and extracted recommendations using well-recognized guideline appraisal tools. Furthermore, recommendations were extracted and reclassified according to a composite grading system.

**Results:**

The search identified 23 guidelines that offered 108 recommendations. Guidelines scored average on AGREE II criteria, with *Scope and Purpose* and *Clarity of Presentation* highest. Only five (22%) guidelines provided high-quality recommendations. The existed clinical recommendations were inconsistent in terms of prophylaxis, diagnosis, and treatment of thromboembolic disease to some extent.

**Conclusion:**

Current guidelines for COVID-19 thromboembolism are generally of low quality, and clinical recommendations on thromboembolism are principally supported by insufficient evidence. There is still an urgent need for more well-designed clinical trials as evidence to prevent adverse events and improve prognosis during COVID-19 treatment.

## 1. Introduction

COVID-19 rapidly spread globally, leading to an ongoing pandemic. Notably, severe patients are at high risk with mortality of approximately 5.44% [1]. A series of retrospective analyses have revealed that one of the most significant poor prognostics in those patients is the development of venous thromboembolism (VTE) [2–4], which may be explained by the damage of the endothelial cells induced by SARS-CoV-2 [5]. Consistently, widespread thrombosis with microangiopathy in patients with COVID-19 according to an autopsy study [6] and alveolar-capillary microthrombi were 9 times as prevalent in patients with COVID-19 as in patients with influenza. Therefore, thromboembolism might be a critical cause of death for severe COVID-19, which should be thought highly of during the diagnosis and treatment.

No specific therapeutic intervention for COVID-19 has yet been established, so supportive care is the most effective aspect of clinical management, supporting the patient's physiology to aid recovery. Optimal provision of supportive care is therefore fundamental both to the wellbeing of individual patients and to securing the confidence of the general population. To enable the provision of the best care, societies and organizations are prone, to sum up, to the experience by a designated guideline or consensus, serving as a better instruction to clinicians.

As is known to all, clinical guidelines could help to assist practitioner and patient decisions about appropriate healthcare for specific clinical circumstances [7]. Widely agreed, rigorous methods now exist for the production and appraisal of clinical guidelines. As the international “gold standard” for guideline development, the Appraisal of Guidelines for Research and Evaluation (AGREE) II tool is a reliable and valid CPG evaluation tool and a foundation upon which to direct CPG development and reporting [8–10]. While Appraisal of Guidelines Research and Evaluation—Recommendations Excellence (AGREE-REX) is a complementary tool for the evaluation of the clinical credibility and implement ability of the guideline recommendation.

With the integrated use of AGREE II and AGREE-REX, this critical appraisal study aimed to assess the quality of the development process and recommendations of thromboembolism guidelines in patients with COVID-19, providing an evidence-based reference for decision-makers to make full use of existed clinical experience. To our knowledge, this is the first critical appraisal of thromboembolism guidelines issued during the COVID-19 pandemic.

## 2. Materials and Methods

We registered this study protocol in the PROSPERO database (CRD42020189419) and reported the results based on the Preferred Reporting Items for Systematic Reviews and Meta-Analyses (PRISMA) statement (eMethods 1).

### 2.1. Guidelines Searches

The search strategies were designed with the assistance of an experienced methodology expert. We searched Medline, Cochrane Library, and Web of Science and the websites of some international organizations and medical societies and gray literature databases, for guidelines focusing on thromboembolism of COVID-19 until October 8, 2020. Additionally, we hand-searched eligible papers' reference lists to ensure a comprehensive review. Details can be found in supplement (eMethods 2 and 3).

### 2.2. Selection of Guidelines

We included documents that provided specific clinical recommendations for diagnosis, prevention, surveillance, and treatment of thromboembolism of COVID-19, which were developed by international organizations, national health institutions, or medical societies. If there were multiple versions of the guidelines, only the latest version would be included. Guidelines only published in English would be eligible. We excluded documents that were concerned about other diseases, such as myocardial infarction, stroke, and cancer, during the COVID-19 pandemic. We also excluded guidelines focused on special populations such as newborns, children, pregnant women, and the elderly. Guidelines were excluded if they were self-organized by a few experts, or if they were written by a hospital institution. Publication types such as reviews, reports, clinical trials, observational studies, commentaries, letters or handbooks, and documents not available in the full text would be excluded. Based on these criteria, two researchers (JL and ZW) screened the documents individually, and any uncertainty was resolved in discussion with a third researcher (MJ). Once the documents were included, we would attempt to retrieve any supplementary files to them to facilitate the article information extraction and evaluation.

### 2.3. Data Extraction

The following information was extracted from each article using a standardized data extraction form: title, full issuing society name, acronym of the guideline, date of publication, country applied, region, target population, type of publication, development method, the strength of recommendation, quality of evidence, version, developers, and the number of developed organizations. Two researchers (JL and ZW) extracted the information individually, and any uncertainty was resolved in discussion with a third researcher (MJ).

### 2.4. Guideline Quality Assessment

A total of four qualified appraisers (JL, ZW, JX, and HL) had been trained through online practice grading and pregrading before the formal assessment. The pregrading was carried out by randomly selecting three eligible guidelines to ensure that each researcher had the same understanding of each item. Using the AGREE II and AGREE-REX tools, each item was scored from 1 (strongly disagree) to 7 (strongly agree). Scores were derived as a percentage of the maximal possible score for each domain, using the following specific formula: (obtained score - minimal possible score)/(maximal possible score - minimal possible score). In AGREE II, we assigned a double weight to the domains of the rigor of development and applicability. A total score greater than 60% would be determined as “recommended,” a score between 30% and 60% as “recommended with modification” and below 30% as “not recommended.” In AGREE-REX, a guideline was classified as “high-quality recommendation” if a total score greater than 70% and “low-quality recommendation” for scores less than 30%. The consistency among the four appraisers was measured using the intraclass correlation coefficient (ICC) with a 95% confidence interval (CI).

### 2.5. Recommendation

Of the 23 eligible guidelines, we extracted recommendations and evidence assessment scales (eTable 1) from 21 guidelines (the clinical recommendations of two guidelines did not sort out one by one, so it was hard to extract). We did not extract recommendations for non-COVID-19 patients. Recommendations, as well as their strength and quality of evidence involving aspects of prevention or monitoring, diagnosis, and treatment of thromboembolism, were extracted. We reclassified the recommendations after reviewing the specific content. Additionally, according to a new comprehensive classification criterion (eTable 2), the strength of recommendation and quality of evidence were redefined so that we could synthesize data and compare the recommendations. The reclassification method designed has been published before [11].

## 3. Results

### 3.1. Characteristics of Included Guidelines

**Figure 1:**
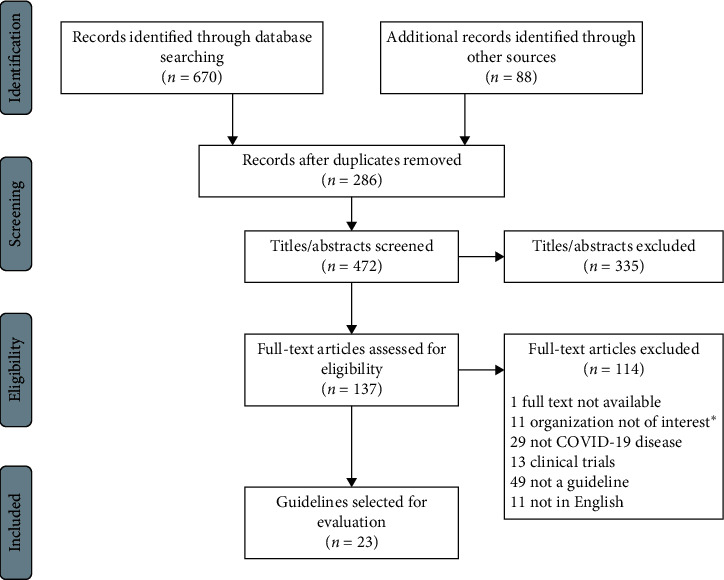
Flowchart of guidelines for thromboembolism in COVID-19 search and selection. Guidelines developed by international organizations, national health institutions, or medical societies would be eligible.

A total of 23 guidelines met the inclusion criteria (Figure 1). The detailed general information of eligible guidelines is shown in Table 1. Eighteen (78%) were published in the first half of the year [12–32]. Four (17%) were developed by international organizations [14–17], 8 (35%) from the Americas [12, 18–21, 29–31], 9 (39%) from Europe [13, 22–26, 32–34], and 2 (9%) from the Asia-Pacific region [27, 28]. Twenty-one (92%) were focused on general population. Six (26%) were self-proclaimed guidelines [12, 16, 17, 22, 27, 31]. Five (22%) had updated versions [12, 14, 18, 27, 32]. Twenty (87%) were developed by medical society [13, 15–31, 33, 34] and 7 (30%) were developed by more than one organization [15, 16, 21, 22, 28, 29, 34]. Eight (35%) were developed by evidence-based approach [12, 14, 15, 17, 20, 27, 28, 31]. Four (17%) provided strength of recommendation [12, 14, 20, 27] and only one (4%) provided quality of evidence [12] (Table 1). Only one (4%) used the Grading of Recommendations Assessment, Development and Evaluation (GRADE) system [27].

### 3.2. Quality Assessment of Guidelines (Figure 2 and eTable 3)

In AGREE II appraisal, *Scope and Purpose* (mean: 65%, range: 33%–86%) and *Clarity of Presentation* (mean: 62%, range: 28%–83%) had higher average scores. The mean score of *Stakeholder Involvement* was 50% (range: 25%–78%) and no public or patients were involved during guideline development. *Rigor of Development* (mean: 39%, range: 20%–72%) and *Applicability* (mean: 38%, range: 7%–58%) had lower average scores. The largest range of scores showed in *Editorial Independence* (mean: 52%, range: 13%–100%). As for the overall evaluation, six (26%) guidelines were recommended, 14 (61%) were recommended with modification, and 3 (13%) were not recommended. The overall agreement was considered good between four appraisers (ICC: 0.87, 95% CI: 0.85–0.89).

In AGREE-REX appraisal, *Clinical Applicability* (mean: 57%, range: 33%–83%) had the highest average scores, and the lowest was *Values and Preferences* (mean: 45%, range: 23%–68%). The largest range of scores showed in *Implementability* (mean: 48%, range: 21%–81%). As for overall evaluation, five (22%) guidelines provided high-quality recommendations and 3 (12%) with low-quality recommendations. The overall agreement was considered good between four appraisers (ICC: 0.85, 95% CI: 0.81–0.88).

### 3.3. Recommendations (Figure 3)

A total of 108 recommendations related to thromboembolism were extracted from 21 guidelines (Table 2). Only 30 (27.8%) provided the strength of recommendation (strong: 19, weak: 11), and only 10 (9.3%) provided the quality of evidence (moderate: 1, very low: 9).

### 3.4. 0Recommendations for Thromboembolism Prevention (eTable 4)

#### 3.4.1. Risk Assessment

Multiple guidelines [13, 15, 17, 20–23, 26, 28, 31] jointly recommended that dynamic and repeated risk assessment for VTE and/or bleeding risk should be conducted for COVID-19 patients to adjust the thromboprophylaxis strategy. Regardless of hospitalized or nonhospitalized patients, it is emphasized that patients should receive pharmacological thromboprophylaxis according to a risk stratification score, unless contraindicated [16, 22–24] (SMW, RCP, CATH, SISET: ungraded). As to the timing of risk assessment, the possibility of thromboembolic disease should be evaluated in the event of rapid deterioration of pulmonary, cardiac, or neurological function, or of sudden, localized loss of peripheral perfusion (NIH [12]: strong, very low; BHS [13], the PERT Consortium [29], CMO [33]: ungraded). On the contrary, JTT [20] (strong, ungraded) recommended a pharmacologic VTE prophylaxis for all hospitalized nonpregnant patients with confirmed or highly suspected COVID-19 regardless of VTE risk assessment score.

#### 3.4.2. Prophylaxis Population

JVS-VL [19] and BSTH&BAHHCT [21] (ungraded) recommended thromboprophylaxis for all patients with COVID-19 or suspected COVID-19. JACC [15], JTH [28], and RCP [22] (ungraded), respectively, recommended that patients with moderate to severe COVID-19, severe COVID-19, and DIC should receive anticoagulant thromboprophylaxis. Thromboprophylaxis strategy differs in various populations. For hospitalized adults with COVID-19, VTE prophylaxis per standard of care for other hospitalized adults is recommended (NIH [12]: strong, very low; CHEST [31], RCP [22]: ungraded). However, there are currently insufficient data to recommend for or against the use of thrombolytics for inpatients (NIH [12]: weak, very low; CATH [16]: ungraded).In terms of nonhospitalized patients with COVID-19, NIH [12] recommended that anticoagulants and antiplatelet therapy should not be initiated for prevention of VTE or arterial thrombosis unless there are other indications (strong, very low).

Moreover, there is currently considerable disagreement as to whether thromboprophylaxis should be administered after discharge. CATH [16] recommended extended VTE prophylaxis after hospital discharge (ungraded). While six guidelines (JTT [20]: weak, ungraded; JTH [28], RCP [22], PCS [25], ISTH [17], BSTH&BAHHCT [21]: ungraded) were generally consistent in determining if a patient has ongoing VTE risk factors at the time of discharge. However, NIH [12] (strong, very low), JTT [20] (weak, ungraded), SFMV [26], and RCP [22] (ungraded) suggest that extended VTE prophylaxis is not necessary for all discharged patients with COVID-19.

#### 3.4.3. Drugs

LMWH is a well-accepted drug in pharmacological prophylaxis. Pharmacological prophylaxis with LMWH is recommended for not only patients hospitalized with COVID-19 by serval guidelines [14, 17, 21, 22, 24–26, 31, 34], but also for patients perceived to have a persistent risk of VTE at the time of discharge (JTH [28]: ungraded; JTT [20]: strong, ungraded). Besides, LMWH is preferable in pharmacological prophylaxis for mild, moderate and severely ill COVID-19 patients assessed to have a risk of VTE (NCCET [27], JTH [28], and BSIM and RBSLM [34]: ungraded) and patients with RRT (RCP [22]: ungraded).

The latest guidelines have put up more drugs for pharmacological prophylaxis. SISET [24], BSTH&BAHHCT [21], ISTH [17], and SFMV [26] (ungraded) consistently recommended the use of UFH or fondaparinux for prophylaxis VTE in COVID-19 hospitalized patients. In addition, RCP [22] (ungraded) recommended that patients already on anticoagulation with a vitamin K antagonist or DOAC can either continue with current anticoagulation or switch to LMWH. ISTH [17] (ungraded) suggests that either LMWH or a DOAC can be used for extended-duration thromboprophylaxis.

JVS-VL [19] (ungraded) recommended that low dose nonnomogram heparin infusion may protect from thrombotic events in patients with ARDS.

However, CHEST [31] (ungraded) objects to the use of antiplatelet agents for VTE prevention in critically ill or acutely ill-hospitalized patients with COVID-19 and gave recommendations on the sequence use of pharmacological prophylaxis, such as LMWH, fondaparinux, UFH, and DOAC. Besides, in critically ill patients with confirmed or highly suspected COVID-19, NCCET [27] (ungraded) and JTT [20] (weak, ungraded) suggest increased prophylactic doses of LMWH.

#### 3.4.4. Dosing and Duration

Talking to the dosing of pharmacological prophylaxis, platelet count, coagulation screen, weight, and renal function are supposed to be considered according to multiple documents [20–22, 26]. Four guidelines [20–22, 26] (SFMV, BSTH&BAHHCT, JTT, and RCP: ungraded) recommended standard dose VTE prophylaxis. ISTH [17] recommended that treatment-dose heparin should not be considered for primary prevention. RCP [22] and ISTH [17] (ungraded) recommended an increased dose of thromboprophylaxis in high-risk patients.

Concerning the duration of thromboprophylaxis, SFMV [26] was recommended for 7 to 14 days, while CATH [16] and ASPHS [30] (ungraded) recommended extending up to 45 days for patients with an elevated risk of VTE and low risk of bleeding. For discharge patients, at least 14 to 28 or 30 days of thromboprophylaxis is advocated by ISTH [17] and RCP [22] (ungraded).

#### 3.4.5. IPC

JTT [20] (ungraded) suggested performing both pharmacologic prophylaxis and IPC in critically ill patients as long as there is no contraindication. Multiple documents [16, 17, 21, 24] recommended that if pharmacological prophylaxis is contraindicated, it is reasonable to consider IPC (JTT [20] and WHO [14]: strong, ungraded).

#### 3.4.6. Self-Management

JACC [15] (ungraded) and JTH [28] (ungraded) encouraged that not only mild or moderate COVID-19 patients but also patients hospitalized or being on discharge should do more physical activity. JTH [28] (ungraded) recommended that mild and moderate COVID-19 patients, especially those with fever and/or gastrointestinal symptoms, should be rehydrated without delay.

### 3.5. Recommendations for Thromboembolism Diagnosis and Monitoring (eTable 5)

#### 3.5.1. Regular Monitoring

JTH [28] (ungraded), JTT [20] (strong, ungraded), and WHO [14] (strong, ungraded) considered clinical conditions should be regularly monitored in COVID-19 patients. SMW [23] (ungraded) believed that many hematological indicators need to be routinely monitored, such as prothrombin time, D-dimers, fibrinogen, and the platelet count. But monitoring antithrombin should only be considered in cases of coagulation dysfunction or heparin resistance due to DIC or sepsis. Especially, the most appropriate diagnostic test is performed within 24 hours in the presence of clinical signs of VTE as advised by CMO [33] (ungraded).

#### 3.5.2. D-Dimer

For the applicable population, NIH [12] (strong, very low) considered that there was not enough evidence to show that nonhospital patients routinely need to be tested for hematology and coagulation indicators, which inpatients usually did (weak, very low). As to its indicative function, D-dimer levels should be monitored and a sudden rise of this marker after an initial decrease in blood with concomitant respiratory failure might suggest VTE (BSIM and RBSLM [34], PCS [25]: ungraded), while some guidelines suggest against its guidance in anticoagulant therapy (JTT [20]: weak, ungraded; BSIM&RBSLM [34]: ungraded), image obtainment (JACC [15] and JVS-VL [19]: ungraded), and diagnostic evaluation (the PERT Consortium [29], ISTH [17], BSTH&BAHHCT [21], SFMV [26], and RCP [22]: ungraded).

#### 3.5.3. Anti-Xa Activity

Anti-Xa activity should be monitored when indicated (SMW [23]: ungraded), except in patients during the use of LMWH (ASHSP [30]: ungraded) or renal replacement therapy (RCP [22]: ungraded). What needed to be distinguished is that using an anti-Xa assay rather than an aPTT to monitor therapeutic UFH in patients whose aPTT was prolonged at baseline (JTT [20]: strong; ungraded) or who exhibited heparin resistance (JTT [20], BSIM, and RBSLM [34]: weak, ungraded). Talking to patients with underlying disease, the obese with an anti-Xa level from 0.2 to 0.5 units/mL may be considered for low-intensity anticoagulation (ASHSP [30]: ungraded).

#### 3.5.4. Imageological Examination

Serval guidelines [12, 17, 19, 22, 26, 28] consistently considered that duplex ultrasonography should be used in case of DVT or PE suspicion (ungraded). Notably, JVS-VL [19] suggested that it should be limited to patients with unilateral limb symptoms (ungraded). For Computed Tomography, confirmation of PE is recommended to be conducted by CTPA (RCP [22], ISTH [17]: ungraded), and SPECT (RCP [22]: ungraded). In particular, venous compression Duplex scan is suggested performing at the admission of ICU patients and then regularly to detect DVT and to prevent its complications (BSTH and BAHHCT [21]: ungraded). For those with right ventricular dysfunction, the diagnosis of PE is important to consider by echocardiography (RCP [22], the PERT Consortium [29], ISTH [17], CMO [33]: ungraded).

### 3.6. Recommendations for Thromboembolism Treatment (eTable 6)

#### 3.6.1. Target Population

Seven guidelines (NIH [12]: strong, very low; others: ungraded) recommended patients with the prior known thrombotic disease to continue their antithrombotic agents. JTH [28] (ungraded) and JTT [20] (strong, ungraded) recommended rescue thrombolytic therapy to critically COVID-19 severe cases with clinical indication.

#### 3.6.2. LMWH

Six guidelines (JTT [20]: weak, ungraded; others: ungraded) considered LMWH should be used in patients with confirmed or suspected VTE. It could also be used in patients at low or moderate risk of bleeding and with no contraindication to antithrombotic drugs (JTH [28], BSIM&RBSLM [34], PCS [25], and CHEST [31]: ungraded). SMW [23] (ungraded) and ASHSP [30] (ungraded) suggested an increased dose should be considered in overweight patients (>100 kg).

#### 3.6.3. UFH

Six guidelines (JTT [20]: strong, ungraded; others: ungraded) considered UFH should be used in patients with creatinine clearance <30 ml/min. As the same as LMWH, SMW [23] (ungraded) and ASHSP [30] (ungraded) suggested an increased dose should be considered in overweight patients (>100 kg). NIH [12] (strong, very low) and SMW [23] (ungraded) both considered that the dosing of LMWH or UFH should be adjusted according to the bleeding risk, which JACC [15] (ungraded) considered insufficient evidence to determine.

Seven guidelines (JTT [20]: weak, ungraded; others: ungraded) considered switching to LMWH in patients taking DOACs or vitamin K antagonist (e.g., warfarin) in case of clinical deterioration. Additionally, five guidelines (JTT [20]: strong, ungraded), others (ungraded, ungraded)) recommended patients who would not be eligible for DOAC therapy before the COVID-19 pandemic or because lopinavir/ritonavir is administered, not to switch to DOAC therapy. JVS-VL [19] (ungraded) considered patients should be transitioned to full dose anticoagulation when no longer at ICU status.

#### 3.6.4. Duration

Five guidelines (ungraded) recommend anticoagulation therapy for a minimum duration of three months, while CATH [16] (ungraded) and CMO [33] (ungraded) considered it reasonable to give extended prophylactic anticoagulation following discharge if there is clinical concern.

## 4. Discussion

As far as we know, this is the first study that critically appraises the scientific evidence and recommendations of thromboembolism guidelines in COVID-19. The quality of existing guidelines on thromboembolism of COVID-19 was low and jagged. Talking to the development of the guideline, although with a clear statement on targeted population and purpose, no public or patients were involved during guideline development in terms of stakeholder involvement, which could reduce the acceptability of guideline promotion. Additionally, attention should be paid to the importance of editorial independence as well. It was shown that financial conflicts of interest were associated with favorable recommendations of drugs and devices in clinical guidelines [36]. Based on the scarce evidence, more transparent recommendations were required. Regarding the development of clinical recommendations, there are also considerable discrepancies. The score ranks last in *Values and Preferences,* in other words, recommendation with a poor representative for multiple populations, which echoes with the above result of AGREE II appraisal. In the process of clinical recommendations extraction, we found that only four guidelines provided the strength of the recommendations, while only one provided quality of evidence. Especially, only a few of them were strong recommendations, and 99% of the recommendations were based on low or ungraded quality because of currently insufficient evidence.

As can be seen from the results, many of the included guidelines were published before July, and most of these guidelines were written specifically to discuss clotting diseases by internationally recognized hematological organizations or were updated versions that specifically renewed recommendations on thromboembolism in the guidelines. Due to the abrupt outbreak of COVID-19, there were many challenges and uncertainties in guiding related clotting diseases. Most of them rarely produced high-quality guidelines or clinical recommendations based on guideline development procedures or on sufficient evidence to support them. As a result, very few of the included guidelines were based on evidence-based methods, and even if they were, they rarely provided the strength of recommendation or quality of evidence. Although the quality of the guidelines was generally low in terms of development methodology or clinical recommendations, the vast majority of the included guidelines considered that they will be updated based on ongoing clinical trial evidence. Therefore, we believed that it was of great benefit to evaluate the quality of current guidelines on thromboembolism of COVID-19 and to extract corresponding clinical recommendations as considerable guidance for clinicians in their practice.

In terms of thromboembolism prevention, the updated guidelines after June illustrated the dosage and duration in more detail and took into account the platelet count, coagulation screen, weight, and renal function. Moreover, the majority of guidelines' recommendations are consistent in the use of IPC and self-management. However, it remained a big divergence in some aspects. Particularly, multiple guidelines recommended conducting a dynamic and repeated risk assessment for thromboembolism and/or bleeding risks for COVID-19 patients and gave the pharmacological thromboprophylaxis according to the risk stratification score. However, JTT [20] emphasized pharmacologic VTE prophylaxis, regardless of VTE risk assessment score (JTT: strong). While as the previous study revealed, a higher occurrence of VTE or mortality is associated with risk assessment score [36, 37], so risk assessment in advance before the pharmacological thromboprophylaxis may be more appropriate. Needless to say, the concept of using LMWH in any hospitalized COVID-19 patient is generally accepted [38]. However, guidelines published after June gave more choices in the thromboprophylaxis drug, such as UFH, fondaparinux. In detail, CHEST [31] emphasized the use of LMWH or fondaparinux over UFH, and it recommended using LMWH, fondaparinux, or UFH over DOAC in acutely ill-hospitalized patients with COVID-19 because concomitant therapy patients received antiviral agents or other investigational treatments that can significantly affect the pharmacodynamics of and thus bleeding risk associated with the DOACs.

Most guidelines believe that it is necessary to routinely monitor patients' clinical symptoms for deterioration and many hematological indicators, especially D-dimer. But the key indicators for monitoring patients with different conditions are various. During the COVID-19 pandemic, patients with prolonged aPTT at baseline and exhibiting heparin resistance should be focused on monitoring anti-Xa levels, while patients with disseminated intravascular coagulation (DIC) or sepsis should be focused on antithrombin indicators. At the same time, most guidelines believe that adjustment on treatment methods or drug dosage should not simply be based on changes in certain hematological indicators. Thus, combining clinical symptoms, laboratory examinations, and imageological examinations for comprehensive judgment is recommended. As for the imaging method, Duplex ultrasonography and CTPA are widely accepted in the screening of DVT or PE. Furthermore, imaging methods should be chosen depending on the patient's actual situation. For patients with severe right ventricular (RV) dysfunction, the diagnosis of PE is important to consider by echocardiography. Diagnostic quality of the scan and less viral spread are key factors that should be taken into consideration in terms of epidemic prevention.

As for thromboembolism treatment with COVID-19, it is recommended that patients with prior known thrombotic should continue their antithrombotic agents. As for COVID-19 severe cases with clinical indication, rescue thrombolytic therapy is recommended by JTH [28] and JTT [20]. Many guidelines advocate the use of LMWH and UFH as antithrombotic drugs unless patients have a contraindication. Based on the fact that LMWH has damage to renal function, it is considered that UFH should be used for patients with renal impairment. For obesity, the dosage of LMWH and UFH needs to be adjusted, which is consistent with recommendations from SMW [23] and ASHSP [30]. Whether using LMWH or UFH, NIH [12] and SMW [23] recommend that it should be based on the patient's bleeding risk for dose adjustment. However, JACC [15] considered insufficient evidence for the issue of dose adjustment, which needed more data of clinical trials to prove. Additionally, several guidelines discussed the use of DOAC, which agreed that if a patient had a worsening of clinical symptoms, DOAC or warfarin were no longer appropriate for treatment and should be switched to LMWH. At last, treatment should be for a minimum duration of three months. Longer durations could be required based on clinical assessment.

Estimates from the US suggested that more than 30% of healthcare was inappropriate or wasteful, and between 70 000 and a third of all deaths occurred annually as a result of medical errors, and that only 55% of needed health services were delivered [39]. The quality of guidelines and recommendations for thromboembolism in COVID-19 were generally low. More high-level evidence focused on this issue should be added as a valuable resource to underline best medical practices and clarify clinical controversies.

## 5. Conclusion

The findings above suggest that guidelines for COVID-19 thromboembolism are generally of low quality, and clinical recommendations on thromboembolism are principally supported by insufficient evidence. There is still an urgent need for more well-designed clinical trials as evidence to guide the practice of front-line clinicians, to effectively prevent adverse events and improve prognosis during COVID-19 treatment.

## Figures and Tables

**Figure 2:**
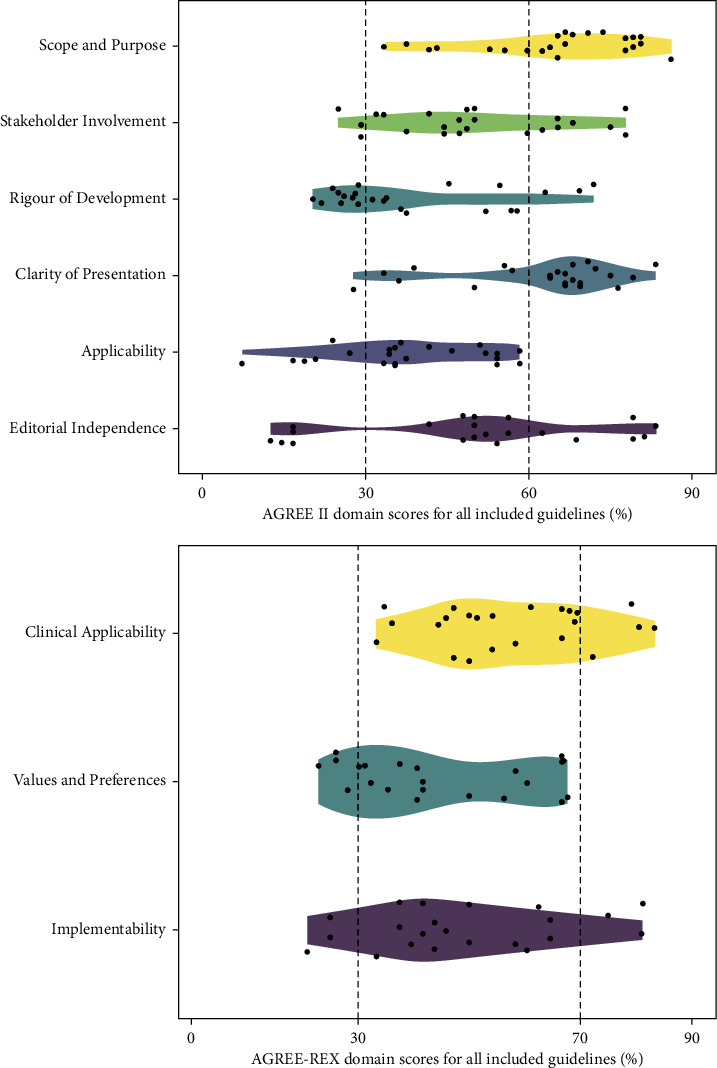
AGREE II and AGREE-REX domain scores for all included guidelines. In domains of AGREE II, a score greater than 60% was classified as “good,” score between 30% and 60% as “moderate,” and below 30% as “poor.” In domains of AGREE-REX, a score greater than 70% was considered as “good,” “moderate” for a score between 30% and 70%, and “poor” for score less than 30%.

**Figure 3:**
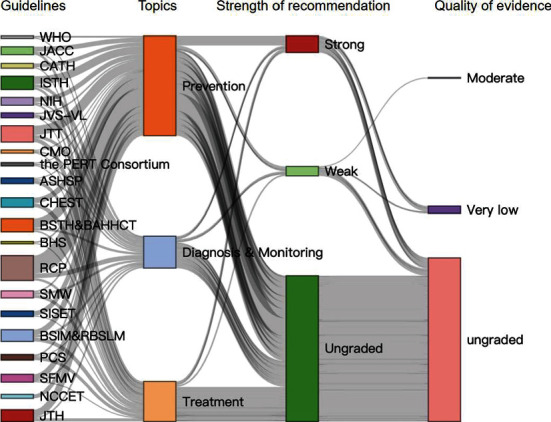
Distribution of strength of recommendations and quality of evidence for thromboembolism. The full name of the guideline abbreviation is as same as those in Table 1.

**Table 1:** The general characteristics of eligible guidelines for thromboembolism in COVID-19.

Title	Issuing society full name	Acronym of the guideline	Date of publication	Country applied	Region	Target population	Type of publication	Development method	Strength of recommendation	Quality of evidence	Grading system	Version	Developers	Number of developed organizations
Clinical Management of COVID-19 [14]	World Health Organization	WHO	2020/5/27	International	International	General population	Others	EB	Yes	No	Not mentioned	Updated	Nonmedical society	1
COVID-19 and Thrombotic or Thromboembolic Disease: Implications for Prevention, Antithrombotic Therapy, and Follow-Up [15]	Journal of the American College of Cardiology	JACC	2020/4/15	International	International	General population	Others	EB	No	No	Not mentioned	First	Medical society	>1
Evidence-Based Practical Guidance for the Antithrombotic Management in Patients with Coronavirus Disease (COVID-19) in 2020 [16]	Brazilian Society of Thrombosis and Hemostasis and the Brazilian Society for Angiology and Vascular Surgery, the International Union of Angiology and the European Venous Forum. Clinical and Applied Thrombosis/Hemostasis	CATH	2020/6/1	International	International	General population	Guidelines	Non-EB	No	No	Not mentioned	First	Medical society	>1
Scientific and Standardization Committee Communication: Clinical Guidance on the Diagnosis, Prevention, and Treatment of Venous Thromboembolism in Hospitalized Patients with COVID-19 [17]	International Society on Thrombosis and Haemostasis	ISTH	2020/5/21	International	International	General population	Guidelines	EB	No	No	Not mentioned	First	Medical society	1
COVID-19 and VTE/Anticoagulation: Frequently Asked Questions [18]	American Society of Hematology	ASH	2020/5/18	USA	North America	General population	Others	Non-EB	No	No	Not mentioned	Updated	Medical society	1
Coronavirus Disease 2019 (COVID-19) Treatment Guidelines [12]	National Institutes of Health	NIH	2020/3/12	USA	North America	General population	Guidelines	EB	Yes	Yes	Not mentioned	Updated	Nonmedical society	1
Practical Diagnosis and Treatment of Suspected Venous Thromboembolism during COVID-19 Pandemic [19]	Journal of Vascular Surgery: Venous and Lymphatic Disorders	JVS-VL	2020/4/14	USA	North America	General population	Others	Non-EB	No	No	Not mentioned	First	Medical society	1
Thromboembolism and Anticoagulant Therapy during the COVID-19 Pandemic-Interim Clinical Guidance from the Anticoagulation Forum [20]	Journal of Thrombosis and Thrombolysis	JTT	2020/5/21	USA	North America	General population	Others	EB	Yes	No	Not mentioned	First	Medical society	1
Diagnosis and Treatment of Pulmonary Embolism during the COVID-19 Pandemic: A Position Paper from the National PERT Consortium [29]	The National Pulmonary Embolism Response Team Consortium	The PERT Consortium	2020/9/24	USA	North America	General population	Others	Non-EB	No	No	Not mentioned	First	Medical society	>1
Practical Considerations in Prevention and Treatment of Venous Thromboembolism in Hospitalized Patients with COVID-19 [30]	American Society of Health-System Pharmacists	ASHSP	2020/7/6	USA	North America	General population	Others	Non-EB	No	No	Not mentioned	First	Medical society	1
Prevention, Diagnosis, and Treatment of VTE in Patients with Coronavirus Disease 2019 CHEST Guideline and Expert Panel Report [31]	American College of Chest Physician	CHEST	2020/9/1	USA	North America	General population	Guidelines	EB	No	No	Not mentioned	First	Medical society	1
Guidance on Diagnosis, Prevention, and Treatment of Thromboembolic Complications in COVID-19: A Position Paper of the Brazilian Society of Thrombosis and Hemostasis and the Thrombosis and Hemostasis Committee of the Brazilian Association of Hematology, Hemotherapy, and Cellular Therapy [21]	The Brazilian Society of Thrombosis and Hemostasis and the Thrombosis and Hemostasis Committee of the Brazilian Association of Hematology, Hemotherapy, and Cellular Therapy	BSTH and BAHHCT	2020/6/30	Brazil	South America	General population	Others	Non-EB	No	No	Not mentioned	First	Medical society	>1
Practical Guidance for the Prevention of Thrombosis and Management of Coagulopathy and Disseminated Intravascular Coagulation of Patients Infected with COVID-19 [13]	British Hematological Society	BHS	2020/3/25	UK	Europe	General population	Others	Non-EB	No	No	Not mentioned	First	Medical society	1
Clinical Guide for the Prevention, Detection, and Management of Thromboembolic Disease in Patients with COVID-19 [22]	Intensive Care Medicine and Intensive Care Society and Association of Anaesthetists and Royal College of Anaesthetists and Royal College of Physicians	RCP	2020/6/19	UK	Europe	General population	Guidelines	Non-EB	No	No	Not mentioned	First	Medical society	>1
COVID-19 Position Statement: The Prevention and Management of Thromboembolism in Hospitalized Patients with COVID-19-Related Disease [33]	Scottish Government's Chief Medical Officer	CMO	2020/7/2	UK	Europe	Hospitalized patients	Others	Non-EB	No	No	Not mentioned	First	Medical society	1
HSE COVID-19: Interim Clinical Guidance–Venous Thromboembolism (VTE) Protocol and Patient Information for Acute Hospitals [35]	Ireland's Health Services	HSE	2020/4/21	Ireland	Europe	General population	Others	Non-EB	No	No	Not mentioned	Updated	Nonmedical society	1
Suggestions for Thromboprophylaxis and Laboratory Monitoring for In-Hospital Patients with COVID-19 [23]	Swiss Medical Weekly	SMW	2020/4/11	Swiss	Europe	General population	Others	Non-EB	No	No	Not mentioned	First	Medical society	1
COVID-19 and Haemostasis: A Position Paper from Italian Society on Thrombosis and Haemostasis (SISET) [24]	Italian Society on Thrombosis and Haemostasis	SISET	2020/4/8	Italy	Europe	General population	Others	Non-EB	No	No	Not mentioned	First	Medical society	1
Belgian Clinical Guidance on Anticoagulation Management in Hospitalized and Ambulatory Patients with COVID-19 [34]	Belgian Society of Internal Medicine and Royal Belgian Society of Laboratory Medicine	BSIM and RBSLM	2020/10/3	Belgium	Europe	General population	Others	Non-EB	No	No	Not mentioned	First	Medical society	>1
Guidance for Anticoagulation Management in Venous Thromboembolism during the Coronavirus Disease 2019 Pandemic in Poland [25]	Polish Cardiac Society	PCS	2020/6/8	Poland	Europe	General population	Others	Non-EB	No	No	Not mentioned	First	Medical society	1
Proposal of the French Society of Vascular Medicine for the Prevention, Diagnosis, and Treatment of Venous Thromboembolic Disease in Outpatients with COVID-19 [26]	French Society of Vascular Medicine	SFMV	2002/4/27	French	Europe	Outpatient	Others	Non-EB	No	No	Not mentioned	First	Medical society	1
Australian Guidelines for the Clinical Care of People with COVID-19 [27]	National COVID-19 Clinical Evidence Taskforce	NCCET	2020/5/28	Australia	Asia-Pacific	General population	Guidelines	EB	Yes	No	GRADE	Updated	Medical society	1
Prevention and Treatment of Venous Thromboembolism Associated with Coronavirus Disease 2019 Infection: A Consensus Statement before guidelines [28]	Journal of Thrombosis and Haemostasis	JTH	2020/4/6	China	Asia-Pacific	General population	Others	EB	No	No	Not mentioned	First	Medical society	>1

**Table 2:** General recommendations for thromboembolism management in COVID-19.

Topic	Type of intervention	Guidelines that provide recommendations	Numbers of recommendations		
			Extracted	Supported by an assessment of strength	Supported by the quality of evidence
Thromboembolism prevention	Risk assessment	BHS, JACC, JTH, SMW, JTT, RCP, ISTH, BSTH&BAHHCT, SFMV, CHEST, NIH, BSIM&RBSLM, the PERT Consortium, CMO, CATH, SISET	7	4 (57%)	2 (29%)
	Prophylaxis population	JVS-VL, BSTH&BAHHCT, NIH, RCP, CHEST, JACC, JTH, CATH, JTT, SFMV, PCS, ISTH	15	5 (33%)	4 (27%)
	Drugs	WHO, RCP, SISET, BSIM&RBSLM, PCS, ISTH, CHEST, BSTH&BAHHCT, SFMV, NCCET, JTH, JTT, JVS-VL	16	3 (19%)	0
	Dosing and duration	RCP, BSTH&BAHHCT, SFMV, JTT, ISTH, NCCET, BSIM&RBSLM, CATH, ASPHS, SISET	28	3 (11%)	0
	IPC	JTT, ISTH, JACC, JTH, WHO, CATH, SISET, BSTH&BAHHCT	3	1 (33%)	0
	Self-management	JACC, JTH	3	0	0

Thromboembolism diagnosis and monitoring	Regular monitoring	JTH, JTT, WHO, SMW, BSTH&BAHHCT, CMO	4	1 (25%)	0
	D-dimer	NIH, JTT, BSIM&RBSLM, JACC, JVS-VL, PCS, the PERT Consortium, ISTH, BSTH&BAHHCT, SFMV, RCP	6	3 (50%)	2 (33%)
	Anti-Xa activity	SMW, JTT, BSIM&RBSLM, ASHSP, RCP	6	2 (33%)	0
	Duplex ultrasonography	JVS-VL, JTH, RCP, ISTH, SFMV, BSTH&BAHHCT	3	0	0
	CTPA(CT)	RCP, ISTH	1	0	0
	Echocardiography	RCP, the PERT Consortium, ISTH, CMO	1	0	0
	Single photon emission computerised tomography (SPECT)	RCP	1	0	0

Thromboembolism treatment	Target population	NIH, JACC, RCP, the PERT Consortium, ASHSP, BSIM&RBSLM, CHEST, JTH, JTT	2	2 (100%)	1 (50%)
	LMWH	JTH, JTT, SISET, PCS, ISTH, BSTH&BAHHCT, BSIM&RBSLM, CHEST, SMW, ASHSP	3	1 (33%)	0
	UFH	JTH, SMW, JTT, ASHSP, BSIM&RBSLM, PCS, NIH, JACC	4	2 (50%)	1 (25%)
	Transition	JTT, RCP, BSIM&RBSLM, PCS, CHEST, BHS, JACC, ISTH, BSTH&BAHHCT, JVS-VL	3	2 (67%)	0
	Duration	RCP, ISTH, CHEST, SFMV, CATH, CMO	2	0	0

The full names of the abbreviation of guidelines are the same as those in Table 1. LMWH: low-molecular-weight heparin; VTE: venous thromboembolism; IPC: intermittent pneumatic compression; UFH: unfractionated heparin.

## Data Availability

Data are available upon author's request.

## Supplementary Materials

Supplementary Materials eMethods 1: PRISMA 2009 Checklist. The page of each section is listed based on the Preferred Reporting Items for Systematic Reviews and Meta-Analyses (PRISMA) statement. eMethods 2: literature search strategies. Search terms used in Medline, Cochrane Library, and Web of Science are listed, respectively. eMethods 3: gray literature search. Except databases mentioned above, some international organizations and medical societies like WHO were searched and the number of included studies is shown. eTable 1: evidence assessment scales used by all included guidelines. The grading system and quality of evidence used in each guideline are shown. eTable 2: a composite grading system for ranking recommendations in guidelines of COVID-19. According to specific definition, the quality of evidence was graded as “high,” “moderate,” “low,” and “very low” and the strength of recommendation was graded as “strong,” “weak,” and “ungraded.” eTable 3: distribution of the strength of recommendation and quality of evidence among clinical recommendation extracted guidelines for thromboembolism in COVID-19. According to the composite grading system, the included guidelines are appraised and the reclassified grade is summarized. eTable 4: AGREE II and AGREE-REX domain scores of included guidelines and overall assessment. There are six domains in AGREE II, which are “Scope and Purpose,” “Stakeholder Involvement,” “Rigor of Development”, “Clarity and Presentation,” “Applicability,” and “Editorial Independence.” And there are three domains in AGREE-REX, which are “Clinical Applicability,” “Values and Preferences,” and “Implementability.” eTable 5: recommendations for thromboembolism prevention in COVID-19. The recommendations for thromboembolism prevention are divided into serval parts: risk assessment, prophylaxis population, drugs, and so on. eTable 6: recommendations for thromboembolism diagnosis and monitoring in COVID-19. The recommendations for thromboembolism diagnosis and monitoring are divided into four parts: regular monitoring, D-dimer, anti-Xa activity, and duplex ultrasonography. eTable 7: recommendations for thromboembolism treatment in COVID-19. The recommendations for thromboembolism Treatment are classified based on different topics: target population, LMWH, UFH, and so on.
