# Supplementary material for: Rigorous Assessment of Guidelines on COVID-19-Related Thrombotic or Thromboembolic Disease: Implications for Clinical Practice of Prevention, Diagnosis, and Treatment
Source: Evid Based Complement Alternat Med. 2021 Sep 24;2021:5513744. doi: 10.1155/2021/5513744 (PMC8476254; doi:10.1155/2021/5513744)
Supplement: Supplementary Materials — eMethods 1: PRISMA 2009 Checklist. The page of each section is listed based on the Preferred Reporting Items for Systematic Reviews and Meta-Analyses (PRISMA) statement. eMethods 2: literature search strategies. Search terms used in Medline, Cochrane Library, and Web of Science are listed, respectively. eMethods 3: gray literature search. Except databases mentioned above, some international organizations and medical societies like WHO were searched and the number of included studies is shown. eTable 1: evidence assessment scales used by all included guidelines. The grading system and quality of evidence used in each guideline are shown. eTable 2: a composite grading system for ranking recommendations in guidelines of COVID-19. According to specific definition, the quality of evidence was graded as “high,” “moderate,” “low,” and “very low” and the strength of recommendation was graded as “strong,” “weak,” and “ungraded.” eTable 3: distribution of the strength of recommendation and quality of evidence among clinical recommendation extracted guidelines for thromboembolism in COVID-19. According to the composite grading system, the included guidelines are appraised and the reclassified grade is summarized. eTable 4: AGREE II and AGREE-REX domain scores of included guidelines and overall assessment. There are six domains in AGREE II, which are “Scope and Purpose,” “Stakeholder Involvement,” “Rigor of Development”, “Clarity and Presentation,” “Applicability,” and “Editorial Independence.” And there are three domains in AGREE-REX, which are “Clinical Applicability,” “Values and Preferences,” and “Implementability.” eTable 5: recommendations for thromboembolism prevention in COVID-19. The recommendations for thromboembolism prevention are divided into serval parts: risk assessment, prophylaxis population, drugs, and so on. eTable 6: recommendations for thromboembolism diagnosis and monitoring in COVID-19. The recommendations for thromboembolism diagnosis and monitoring are [file 5513744.f1.docx]

**Supplementary Online Content**

1. **eMethods 1.** PRISMA 2009 Checklist
2. **eMethods 2.** Literature Search Strategies
3. **eMethods 3.** Gray Literature Search
4. **eTable 1.** Evidence assessment scales used by all included guidelines
5. **eTable 2.** A composite grading system for ranking recommendations in guidelines of COVID-19
6. **eTable 3.** Distribution of the strength of recommendation and quality of evidence among clinical recommendation extracted guidelines for thromboembolism in COVID-19
7. **eTable 4.** AGREE II & AGREE-REX domain scores of included guidelines and overall assessment
8. **eTable 5.** Recommendations for Thromboembolism Prevention in COVID-19
9. **eTable 6.** Recommendations for Thromboembolism Diagnosis & Monitoring in COVID-19
10. **eTable 7.** Recommendations for Thromboembolism Treatment in COVID-19

**eMethods 1. PRISMA 2009 Checklist**

| **Section/topic** | **#** | **Checklist item** | **Reported on page #** |
| --- | --- | --- | --- |
| **TITLE** | | |  |
| Title | 1 | Identify the report as a systematic review, meta-analysis, or both. | Page 1 |
| **ABSTRACT** | | |  |
| Structured summary | 2 | Provide a structured summary including, as applicable: background; objectives; data sources; study eligibility criteria, participants, and interventions; study appraisal and synthesis methods; results; limitations; conclusions and implications of key findings; systematic review registration number. | Page 3 |
| **INTRODUCTION** | | |  |
| Rationale | 3 | Describe the rationale for the review in the context of what is already known. | Page 6 |
| Objectives | 4 | Provide an explicit statement of questions being addressed with reference to participants, interventions, comparisons, outcomes, and study design (PICOS). | Page 6 |
| **METHODS** | | |  |
| Protocol and registration | 5 | Indicate if a review protocol exists, if and where it can be accessed (e.g., Web address), and, if available, provide registration information including registration number. | Page 7 |
| Eligibility criteria | 6 | Specify study characteristics (e.g., PICOS, length of follow-up) and report characteristics (e.g., years considered, language, publication status) used as criteria for eligibility, giving rationale. | Page 7 |
| Information sources | 7 | Describe all information sources (e.g., databases with dates of coverage, contact with study authors to identify additional studies) in the search and date last searched. | Page 7 |
| Search | 8 | Present full electronic search strategy for at least one database, including any limits used, such that it could be repeated. | eMethods 2&3 |
| Study selection | 9 | State the process for selecting studies (i.e., screening, eligibility, included in systematic review, and, if applicable, included in the meta-analysis). | Page 7 |
| Data collection process | 10 | Describe method of data extraction from reports (e.g., piloted forms, independently, in duplicate) and any processes for obtaining and confirming data from investigators. | Page 7 |
| Data items | 11 | List and define all variables for which data were sought (e.g., PICOS, funding sources) and any assumptions and simplifications made. | Page 8 |
| Risk of bias in individual studies | 12 | Describe methods used for assessing risk of bias of individual studies (including specification of whether this was done at the study or outcome level), and how this information is to be used in any data synthesis. | Page 8 |
| Summary measures | 13 | State the principal summary measures (e.g., risk ratio, difference in means). | Page 8 |
| Synthesis of results | 14 | Describe the methods of handling data and combining results of studies, if done, including measures of consistency (e.g., I^2^) for each meta-analysis. | Page 8 |

Page 1 of 2

| **Section/topic** | **#** | **Checklist item** | **Reported on page #** |
| --- | --- | --- | --- |
| Risk of bias across studies | 15 | Specify any assessment of risk of bias that may affect the cumulative evidence (e.g., publication bias, selective reporting within studies). | Page 7 |
| Additional analyses | 16 | Describe methods of additional analyses (e.g., sensitivity or subgroup analyses, meta-regression), if done, indicating which were pre-specified. | NA |
| **RESULTS** | | |  |
| Study selection | 17 | Give numbers of studies screened, assessed for eligibility, and included in the review, with reasons for exclusions at each stage, ideally with a flow diagram. | Page 8 |
| Study characteristics | 18 | For each study, present characteristics for which data were extracted (e.g., study size, PICOS, follow-up period) and provide the citations. | Page 8  Table 1 |
| Risk of bias within studies | 19 | Present data on risk of bias of each study and, if available, any outcome level assessment (see item 12). | Page 9 |
| Results of individual studies | 20 | For all outcomes considered (benefits or harms), present, for each study: (a) simple summary data for each intervention group (b) effect estimates and confidence intervals, ideally with a forest plot. | Table 2, eTable 5-7 |
| Synthesis of results | 21 | Present results of each meta-analysis done, including confidence intervals and measures of consistency. | NA |
| Risk of bias across studies | 22 | Present results of any assessment of risk of bias across studies (see Item 15). | eTable 4 |
| Additional analysis | 23 | Give results of additional analyses, if done (e.g., sensitivity or subgroup analyses, meta-regression [see Item 16]). | NA |
| **DISCUSSION** | | |  |
| Summary of evidence | 24 | Summarize the main findings including the strength of evidence for each main outcome; consider their relevance to key groups (e.g., healthcare providers, users, and policy makers). | Page 13-15 |
| Limitations | 25 | Discuss limitations at study and outcome level (e.g., risk of bias), and at review-level (e.g., incomplete retrieval of identified research, reporting bias). | Page 16 |
| Conclusions | 26 | Provide a general interpretation of the results in the context of other evidence, and implications for future research. | Page 16 |
| **FUNDING** | | |  |
| Funding | 27 | Describe sources of funding for the systematic review and other support (e.g., supply of data); role of funders for the systematic review. | Page 17 |

*From:*  Moher D, Liberati A, Tetzlaff J, Altman DG, The PRISMA Group (2009). Preferred Reporting Items for Systematic Reviews and Meta-Analyses: The PRISMA Statement. PLoS Med 6(6): e1000097. doi:10.1371/journal.pmed1000097

For more information, visit: **www.prisma-statement.org**.

Page 2 of 2

**eMethods 2. Literature Search Strategies**

**MEDLINE (PubMed) Search Strategy**

#1 hemostasis[MeSH Terms] (112,953)

#2 blood clot*[All Fields] (40,721)

#3 coagulat*[All Fields] (164,329)

#4 haemosta*[All Fields] (17,694)

#5 thromb*[All Fields] (540,411)

#6 antithrombin*[All Fields] (31,794)

#7 #1 OR #2 OR #3 OR #4 OR #5 OR #6 (687,906)

#8 Guideline[Publication Type] (33,887)

#9 guid*[Title/Abstract] (771,582)

#10 consensus*[Title/Abstract] (161,911)

#11 recommendat*[Title/Abstract] (255,152)

#12 best practice*[Title/Abstract] (25,455)

#13 #8 OR #9 OR #10 OR #11 OR #12 (1,106,839)

#14 #7 AND #13 (33,152)

#15 COVID-19[Title/Abstract] OR SARS2[Title/Abstract] OR coronavirus[Title/Abstract] OR SARS-CoV-2[Title/Abstract] (26,832)

#16 #14 AND #15 (58)

**Cochrane Library Search Strategy**

#1 MeSH descriptor: [Severe Acute Respiratory Syndrome] explode all trees (107)

#2 (coronavirus disease OR "2019-nCoV" OR SARS2):ti,ab,kw (179)

#3 MeSH descriptor: [SARS Virus] explode all trees (9)

#4 ("COVID-19 virus" OR "SARS-CoV-2" OR 2019 novel coronavirus OR severe acute respiratory syndrome coronavirus 2):ti,ab,kw (25)

#5 {OR #1-#4} (227)

#6 ("guid*" OR "consensus*" OR "recommendat*" OR "best practice*" OR "CPG" OR "CPGs"):ti,ab,kw (44604)

#7 {AND #5-#6} (12)

**Web of Science Search Strategy**

(TS=("COVID-19" OR "2019-nCoV" OR "SARS2") OR TS=("COVID-19 virus" OR "SARS-CoV-2" OR "2019 novel coronavirus" OR "severe acute respiratory syndrome coronavirus 2" OR "coronavirus")) AND (TS=("guid*" OR "consensus*" OR "recommendat*" OR "best practice*" OR "CPG" OR "CPGs")) AND (TS=("hemosta*" OR "blood clot*" OR "coagulat*" OR "haemosta*" OR "thromb*" OR "antithrombin*")) (date=2019-2020) (55)

**eMethods 3. Gray Literature Search**

1. Search Terms: (guideline) AND (COVID-19 OR 2019-nCoV OR SARS-CoV-2) AND ("hemostasis" OR "haemostasis" OR "thrombosis" OR "coagulation")
2. Sources:
3. Webpages of relevant organizations
4. [World Health Organization (WHO) Country & Technical Guidance - Coronavirus disease (COVID-19)](https://www.who.int/emergencies/diseases/novel-coronavirus-2019/technical-guidance) (1)
5. [Centers for Disease Control and Prevention (CDC) Guidance on COVID-19](https://www.cdc.gov/coronavirus/2019-ncov/hcp/index.html) (0)
6. [the FDA - food and drug administration](https://www.fda.gov/emergency-preparedness-and-response/counterterrorism-and-emerging-threats/coronavirus-disease-2019-covid-19) (0)
7. [European Respiratory Society](https://www.ersnet.org/covid-19-guidelines-and-recommendations-directory) (0)
8. [NHS England and NHS Improvement](https://www.england.nhs.uk) (0)
9. [Society of Critical Care Medicine (SCCM)](https://covid19.sccm.org) (0)
10. [Massachusetts General Hospital](https://www.massgeneral.org/news/coronavirus/treatment-guidance/inpatient-care-recommendations) (2)
11. [India NCDC](https://ncdc.gov.in/index1.php?page=1&ipp=All&lang=1&level=1&sublinkid=703&lid=550) (0)
12. [European CDC](https://www.ecdc.europa.eu/en/coronavirus/guidance-and-technical-reports) (0)
13. [Government of Canada](https://www.canada.ca/en/public-health/services/diseases/2019-novel-coronavirus-infection/clinical-management-covid-19.html) (0)
14. [CHEST](https://www.chestnet.org) (0)
15. Gray literature databases
16. [Guidelines International Network (G-I-N)](http://www.g-i-n.net/library/international-guidelines-library) (0)
17. [ECRI Guidelines Trust](https://guidelines.ecri.org/) (1)
18. [National Institute for Health and Clinical Excellence (NICE)](http://www.nice.org.uk/) (0)
19. [Scottish Intercollegiate Guidelines Network (SIGN)](http://www.sign.ac.uk/index.html) (0)
20. [The National Health and Medical Research Council (NHMRC) Guidelines](https://www.nhmrc.gov.au/health-advice/guidelines) (0)
21. [Pan American Health Organization (PAHO) guidelines](https://www.paho.org/en/guidelines?topic=All&d%5Bmin%5D=&d%5Bmax%5D=&page=2) (0)
22. [NATIONAL COVID-19 CLINICAL EVIDENCE TASKFORCE](https://covid19evidence.net.au/) (1)
23. [société de pneumologie de langue française (splf)](http://splf.fr/centre-de-documentation-covid-19/) (0)

Citation index or other web sites (5)

**eTable 1.** Evidence assessment scales used by all included guidelines

| **Guideline*** | **Grading System** | **Strength of Recommendation** | **Quality of Evidence** |
| --- | --- | --- | --- |
| **NCCET^20^** | Grading of Recommendations Assessment Development and Evaluation (GRADE) | **Recommendation for (Green):** A strong recommendation is given when there is high-certainty evidence showing that the overall benefits of the intervention are clearly greater than the disadvantages. This means that all, or nearly all, patients will want the recommended intervention.  **Recommendation against (Green + Red):** A strong recommendation against the intervention is given when there is high-certainty evidence showing that the overall disadvantages of the intervention are clearly greater than the benefits. A strong recommendation is also used when the examination of the evidence shows that an intervention is not safe.  **Conditional Recommendation for (Yellow):** A conditional recommendation is given when it is considered that the benefits of the intervention are greater than the disadvantages, or the available evidence cannot rule out a significant benefit of the intervention while assessing that the adverse effects are few or absent. This recommendation is also used when patient preferences vary.  **Conditional Recommendation against (Yellow + Red):** A conditional recommendation is given against the intervention when it is judged that the disadvantages of the intervention are greater than the benefits, but where this is not substantiated by strong evidence. This recommendation is also used where there is strong evidence of both beneficial and harmful effects, but where the balance between them is difficult to determine. Likewise, it is also used when patient preferences vary.  **Consensus Recommendation (Purple):** A consensus recommendation can be given for or against the intervention. This type of recommendation is used when there is not enough evidence to give an evidence-based recommendation, but the panel still regards it as important to give a recommendation. | **High:** We are very sure that the true effect is close to the estimated effect.  **Moderate:** We are moderately sure of the estimated effect. The true effect is probably close to this one, but there is a possibility that it is significantly different.  **Low:** We have limited confidence in the estimated effect. The true effect may be significantly different from the estimated effect.  **Very low:** We have very little confidence in the estimated effect. The true effect is likely to be significantly different from that estimated effect. |
|  |  |  |  |
| **NIH^11^** | Not mention | **A:** Strong recommendation for  the statement.  **B:** Moderate recommendation  for the statement.  **C:** Optional recommendation  for the statement. | **I:** One or more randomized trials with clinical outcomes and/or validated laboratory endpoints.  **II:** One or more well designed, nonrandomized trials or observational cohort studies.  **III:** Expert opinion. |
|  |  |  |  |
| **JTT^17^** | Not mention | **we recommend-**practices for which there is the strongest evidence and/or nearly unanimous expert opinion    **we suggest -**practices for which there is less strong evidence and/or lack of consensus  **is reasonable -**practices for which little to no evidence exists and/or there is lack of consensus | Not mention |
|  |  |  |  |
| **WHO^14^** | Not mention | **The GREEN symbol** denotes a strong recommendation or a best practice statement in favour of an intervention.  **The RED symbol** denotes a recommendation or a best practice statement against an Intervention.  **The YELLOW symbol** denotes a conditional recommendation in favour of an intervention, or a recommendation where special care is required in implementation. | Not mention |

*: The full name of the abbreviation is as same as those in Table 1.

**eTable 2. A** **composite grading system for ranking recommendations inguidelines of COVID 19**

| **Category** | **Grade** | **Definition** |
| --- | --- | --- |
| **Quality of evidence** | **High** | Randomized controlled trials without important limitations, or meta-analysis |
|  | **Moderate** | Randomized controlled trials with important limitations, or upgraded observational studies |
|  | **Low** | Non-randomized studies, cohort or case-control studies, case series |
|  | **Very low** | Expert opinion |
|  |  |  |
| **Strength of recommendation** | **Strong** | Recommendation can apply to most patients in many circumstances |
|  | **Weak** | The best action may differ depending on circumstances or patients’ or societal values |
|  | **Ungraded** | Insufficient evidence on which to formulate a recommendation. The advantages and disadvantages of the procedure/treatment are equivalent. |

**eTable 3. Distribution of the strength of recommendation and quality of evidence among clinical recommendation extracted guidelines for thromboembolism in COVID-19***

| **Guideline** | **Number of Recommendations** | **Strength of Recommendation, n(%)** | | | **Quality of Evidence, n(%)** | | | |  |
| --- | --- | --- | --- | --- | --- | --- | --- | --- | --- |
|  |  | **Strong** | **Weak** | **Ungraded** | **High** | **Moderate** | **Low** | **Very low** | **Ungraded** |
| NCCET^20^ | 2 | 0 | 0 | 2(100.0) | 0 | 0 | 0 | 0 | 2(100.0) |
| BHS^12^ | 8 | 0 | 0 | 8(100.0) | 0 | 0 | 0 | 0 | 8(100.0) |
| NIH^11^ | 17 | 12(70.6) | 5(29.4) | 0 | 0 | 1(5.9) | 0 | 16(94.1) | 0 |
| JACC^13^ | 15 | 0 | 0 | 15(100.0) | 0 | 0 | 0 | 0 | 15(100.0) |
| JVS-VL^16^ | 9 | 0 | 0 | 9(100.0) | 0 | 0 | 0 | 0 | 9(100.0) |
| JTH^21^ | 17 | 0 | 0 | 17(100.0) | 0 | 0 | 0 | 0 | 17(100.0) |
| SMW^19^ | 10 | 0 | 0 | 10(100.0) | 0 | 0 | 0 | 0 | 10(100.0) |
| JTT^17^ | 37 | 21(56.8) | 14(37.8) | 2(5.4) | 0 | 0 | 0 | 0 | 37(100.0) |
| WHO^14^ | 3 | 3(100.0) | 0 | 0 | 0 | 0 | 0 | 0 | 3(100.0) |
| Total, n (%) | 118 | 36(30.5) | 19(16.1) | 63(59.5) | 0 | 1(0.8) | 0 | 16(13.6) | 101(85.6) |

*: The full names of the abbreviation of guidelines are as same as those in Table 1.

Data are presented as n (%)

**eTable 4. AGREE II & AGREE-REX domain scores of included guidelines and overall assessment**

| **Guideline*** | **AGREE II** | | | | | | |  | **AGREE-REX** | | | |
| --- | --- | --- | --- | --- | --- | --- | --- | --- | --- | --- | --- | --- |
|  | **Scope and Purpose** | **Stakeholder Involvement** | **Rigor of Development** | **Clarity and Presentation** | **Applicability** | **Editorial Independence** | **Overall Assessment** |  | **Clinical Applicability** | **Values and Preferences** | **Implementability** | **Overall Assessment** |
| **ASH^15^** | 63 | 38 | 38 | 67 | 35 | 17 | Recommended with modification |  | 54 | 32 | 46 | Moderate quality recommendation |
| **NCCET^20^** | 78 | 65 | 58 | 71 | 58 | 48 | Recommended |  | 68 | 50 | 65 | Moderate quality recommendation |
| **HSE^18^** | 42 | 42 | 26 | 39 | 24 | 13 | Not recommended |  | 36 | 26 | 25 | Low quality recommendation |
| **BHS^12^** | 60 | 33 | 36 | 69 | 38 | 15 | Recommended with modification |  | 47 | 31 | 38 | Moderate quality recommendation |
| **NIH^11^** | 86 | 78 | 72 | 83 | 58 | 56 | Recommended |  | 83 | 68 | 75 | High quality recommendation |
| **JACC^13^** | 81 | 78 | 63 | 75 | 51 | 54 | Recommended |  | 79 | 67 | 65 | High quality recommendation |
| **JVS-VL^16^** | 64 | 44 | 29 | 68 | 36 | 48 | Recommended with modification |  | 54 | 35 | 33 | Moderate quality recommendation |
| **JTH^21^** | 81 | 75 | 69 | 79 | 54 | 69 | Recommended |  | 81 | 67 | 63 | High quality recommendation |
| **SMW^19^** | 38 | 25 | 20 | 28 | 7 | 83 | Not recommended |  | 35 | 26 | 25 | Low quality recommendation |
| **JTT^17^** | 71 | 47 | 33 | 68 | 34 | 81 | Recommended with modification |  | 67 | 28 | 38 | Moderate quality recommendation |
| **WHO^14^** | 78 | 68 | 57 | 76 | 54 | 56 | Recommended |  | 69 | 67 | 81 | High quality recommendation |
| **Total #** | **67 (38,86)** | **54 (25,78)** | **46 (20,72)** | **66 (28,83)** | **41 (7,58)** | **49 (13,83)** |  |  | **61 (35,83)** | **45 (26,68)** | **50 (25,81)** |  |

*: The full name of the abbreviation is as same as those in Table 1.

#: Data are presented as mean and range.

**eTable 5.** **Recommendations for Thromboembolism Prevention in COVID-19***

| **Intervention** | **Recommendation** | **Supporting Guidelines** | **Strength of Recommendation#** | **Quality of evidence#** |
| --- | --- | --- | --- | --- |
| Risk assessment | For hospitalized COVID-19 patients, the possibility of thromboembolic disease should be evaluated in the event of rapid deterioration of pulmonary, cardiac, or neurological function, or of sudden, localized loss of peripheral perfusion | BHS，NIH | Strong: NIH  Ungraded: BHS | Very low: NIH  Ungraded: BHS |
|  | Dynamic and repeated risk assessment for VTE and/or bleeding risks should be conducted for COVID-19 patients to adjust thromboprophylaxis strategy | BHS, JACC, JTH, SMW, JTT | Strong: JTT  Ungraded: BHS, JACC, JTH, SMW | Ungraded: BHS, JACC, JTH, SMW, JTT |
|  | Avoiding the need to reassess VTE risk when a patient has a change in status | JVS-VL | Ungraded | Ungraded |
|  | Pharmacologic prophylaxis could be considered after risk assessment on patients who are at low risk for bleeding and high risk for VTE | NIH，JACC | Weak: NIH  Ungraded: JACC | Moderate: NIH  Ungraded: JACC |
|  | We recommend pharmacologic VTE prophylaxis for all hospitalized non-pregnant patients with confirmed or highly suspected COVID-19, regardless of VTE risk assessment score | JTT | Strong | Ungraded |
|  | All in-hospital COVID-19 patients should receive pharmacological thromboprophylaxis according to a risk stratification score, unless contraindicated | SMW | Ungraded | Ungraded |
| Prophylaxis population | Hospitalized adults with COVID-19 should receive VTE prophylaxis per the standard of care for other hospitalized adults | NIH | Strong | Very low |
|  | All patients with COVID-19 or suspected COVID-19 should be treated with thromboprophylaxis. | JVS-VL | Ungraded | Ungraded |
|  | For patients with moderate or severe COVID-19 and in DIC but without overt bleeding, prophylactic anticoagulation should be administered | JACC | Ungraded | Ungraded |
|  | We suggest that a multidisciplinary discussion occur at or near the time of discharge to determine if a patient has ongoing VTE risk factors | JTH, JTT | Weak: JTT  Ungraded: JTH | Ungraded |
|  | Mild and moderate COVID-19 patients perceived to have a persistent risk of VTE at the time of discharge, a prolonged outpatient VTE prophylaxis care should be considered with LMWH over DOAC use | JTH | Ungraded | Ungraded |
|  | For non-hospitalized patients with COVID-19, anticoagulants and antiplatelet therapy should not be initiated for prevention of venous thromboembolism (VTE) or arterial thrombosis unless there are other indications | NIH | Strong | Very low |
|  | There are currently insufficient data to recommend for or against the use of thrombolytics or increasing anticoagulant doses for VTE prophylaxis in hospitalized COVID-19 patients outside the setting of a clinical trial | NIH | Weak | Very low |
|  | All severe and critically ill COVID-19 patients have a high risk of VTE, so prevention of VTE is strongly recommended in absence of contraindication. | JTH | Ungraded | Ungraded |
|  | We suggest that extended VTE prophylaxis is not necessary for all patients with COVID-19 who are being discharged from the hospital. | NIH, JTT | Strong: NIH  Weak: JTT | Very low: NIH  Ungraded: JTT |
| Drugs | patients perceived to have a persistent risk of VTE at the time of discharge, we recommend use LMWH | JTH, JTT | Strong: JTT  Ungraded: JTH | Ungraded |
|  | Mild and moderate COVID-19 patients assessed to have a high or moderate risk of VTE, LMWH is recommended preferably | NCCET, JTH | Ungraded | Ungraded |
|  | For critically ill patients with confirmed or highly suspected COVID-19, we suggest increased prophylactic doses of LMWH | NCCET, JTT | Weak: JTT  Ungraded: NCCET | Ungraded |
|  | In patients hospitalized with COVID-19, use pharmacological prophylaxis, such as LMWH | WHO | Strong | Ungraded |
|  | In patients with ARDS, low dose non-nomogram heparin infusion may protect from thrombotic events | JVS-VL | Ungraded | Ungraded |
| Medication dose | For all non-critically ill hospitalized patients with confirmed or highly suspected COVID-19 or patients that are improving and transferring out of the ICU to the medical ward, we recommend standard dose VTE prophylaxis. | JTT | Ungraded | Ungraded |
|  | For all non-critically ill hospitalized patients, dose adjustments for renal function or extremes of weight should follow product labeling and/or institutional protocols. | JTT | Strong | Ungraded |
|  | patients with moderate COVID-19 and severe acute kidney disease is present, enoxaparin 20mg once daily or dalteparin 2500 IU once daily may be used | NCCET | Ungraded | Ungraded |
|  | Patients with severe or critical COVID-19 and severe acute kidney disease, enoxaparin 40 mg once daily or dalteparin 5000 IU once daily may be used | NCCET | Ungraded | Ungraded |
|  | For critically ill patients with confirmed or highly suspected COVID-19, we suggest use enoxaparin 40 mg subcutaneous twice daily, enoxaparin 0.5 mg/kg subcutaneous twice daily, heparin 7500 units subcutaneous three times daily, or low-intensity heparin infusion | JTT | Weak | Ungraded |
|  | For moderate or critically ill patients with confirmed or highly suspected COVID-19, we suggest use enoxaparin 40 mg twice daily or dalteparin 5000 IU twice daily | NCCET | Ungraded | Ungraded |
| IPC | In critically ill patients, it is reasonable to employ both pharmacologic and IPC as long as no contraindication | JTT | Ungraded | Ungraded |
|  | If pharmacological prophylaxis is contraindicated, it is reasonable to consider IPC | JACC, JTH, JTT, WHO | Strong: JTT, WHO  Ungraded: JACC, JTH | Ungraded |
| Self-management | For patients who were admitted and are now being discharged for COVID-19, ambulation and physical activity should be encouraged | JACC | Ungraded | Ungraded: |
|  | For mild and moderate COVID-19 patients, increased mobility should be encouraged. | JACC, JTH | Ungraded | Ungraded |
|  | Mild and moderate COVID-19 patients, especially those with fever and/or gastrointestinal symptoms, should be rehydrated without delay | JTH | Ungraded | Ungraded |

*: The full names of the abbreviation of guidelines are as same as those in Table 1.

#: Strength of recommendation and quality of evidence were harmonized according the composite grading system shown in eTable2.

Other abbreviation: DOAC: direct oral anticoagulant, LMWH: Low-molecular-weight heparin, VTE: Venous thromboembolism, IPC: intermittent pneumatic compression

**eTable 6.** **Recommendations for Thromboembolism Diagnosis & Monitoring in COVID-19***

| **Intervention** | **Recommendation** | **Supporting Guidelines** | **Strength of Recommendation#** | **Quality of evidence#** |
| --- | --- | --- | --- | --- |
| Regular monitoring | Any change of the clinical condition should be regularly monitored in COVID-19 patients. | JTH, JTT, WHO | Strong: JTT, WHO  Ungraded: JTH | Ungraded |
|  | prothrombin time, D-dimers, fibrinogen, the platelet count, lactate dehydrogenase (LDH), creatinine and alanine aminotransferase (ALT) | SMW | Ungraded | Ungraded |
|  | Antithrombin need not be monitored, but could be considered in cases of disseminated intravascular coagulation or sepsis-induced coagulopathy or heparin resistance. | SMW | Ungraded | Ungraded |
| D-dimer | In non-hospitalized patients with COVID-19, there are currently no data to support the measurement. | NIH | Strong | Very low |
|  | In hospitalized patients with COVID-19, hematologic and coagulation parameters are commonly measured | NIH | Weak | Very low |
|  | suggest against daily monitoring of D-dimer for the purpose of guiding anticoagulant therapy | JTT | Weak | Ungraded |
|  | Elevated D-dimer should not be a determinant in the decision to obtain imaging. | JACC, JVS-VL | Ungraded | Ungraded |
| Anti-Xa activity | should be monitored when indicated | SMW | Ungraded | Ungraded |
|  | using an anti-Xa assay rather than an aPTT to monitor therapeutic UFH in patients with COVID-19 whose aPTT is prolonged at baseline. | JTT | Strong | Ungraded |
|  | using an anti-Xa assay rather than an aPTT to monitor therapeutic UFH in patients with COVID-19 who exhibit heparin resistance | JTT | Weak | Ungraded |
| Duplex ultrasonography | Be used in case of DVT or PE suspicion | JVS-VL, JTH | Ungraded | Ungraded |
|  | should be limited to patients with unilateral limb symptoms. | JVS-VL | Ungraded | Ungraded |

*: The full names of the abbreviation of guidelines are as same as those in Table 1.

#: Strength of recommendation and quality of evidence were harmonized according the composite grading system shown in eTable2.

Other abbreviation: aPTT: activated partial thromboplastin time, DVT: deep vein thrombosis, PE: pulmonary embolism, UFH: Unfractionated heparin

**eTable 7. Recommendations for Thromboembolism Treatment in COVID-19***

| **Intervention** | **Recommendation** | **Supporting Guidelines** | **Strength of Recommendation#** | **Quality of evidence#** |
| --- | --- | --- | --- | --- |
| Target population | patients for prior known thrombotic disease, they should continue their antithrombotic agents as recommended. | NIH, JACC | Strong: NIH  Ungraded: JACC | Very low: NIH  Ungraded: JACC |
|  | rescue thrombolytic therapy is recommended in critically COVID-19 severe cases with clinical indication. | JTH, JTT | Strong: JTT  Ungraded: JTH | Ungraded |
| LMWH | patients with confirmed or suspected VTE | JTH, JTT | Weak: JTT Ungraded: JTH | Ungraded: JTH, JTT |
|  | patients at low or moderate risk of bleeding and with no contraindication to antithrombotic drugs. | JTH | Ungraded | Ungraded |
|  | An increased dose should be considered in overweight patients (>100 kg) | SMW | Ungraded | Ungraded |
| UFH | patients with creatinine clearance <30 ml/min | JTH, SMW, JTT | Strong: JTT  Ungraded: JTH, SMW | Ungraded |
|  | An increased dose should be considered in overweight patients (>100 kg) | SMW | Ungraded | Ungraded |
|  | Intermediate or therapeutic dosing of LMWH or UFH should be considered, according to the bleeding risk. | NIH, SMW | Strong: NIH  Ungraded: SMW | Very low: NIH  Ungraded: SMW |
|  | There is insufficient data to consider routine therapeutic or intermediate-dose parenteral anticoagulation with UFH or LMWH. | JACC | Ungraded | Ungraded |
| Transition | patients who would not be eligible for DOAC therapy prior to the COVID-19 pandemic should not be switched to DOAC therapy. | JTT | Strong | Ungraded |
|  | Consider switching to LMWH in patients taking DOACs or vitamin K antagonist (e.g warfarin) in case of clinical deterioration. | BHS, JACC, JTT | Weak: JTT  Ungraded: BHS, JACC | Ungraded |
|  | Patients treated with low dose anticoagulation protocols should be transitioned to full  dose anticoagulation when no longer ICU status. | JVS-VL | Ungraded | Ungraded |

*: The full names of the abbreviation of guidelines are as same as those in Table 1.

#: Strength of recommendation and quality of evidence were harmonized according the composite grading system shown in eTable2.

Other abbreviation: DOAC: direct oral anticoagulant, ICU: Intensive care unit, LMWH: Low-molecular-weight heparin, VTE: Venous thromboembolism, UFH: Unfractionated heparin
